# Evaluation of the Safety and Tolerability of L-Tyrosine Supplementation in Healthy Adult Men: A Randomized Crossover Trial

**DOI:** 10.3390/nu18122020

**Published:** 2026-06-21

**Authors:** Hideki Matsumoto, Naoki Miura, Masaki Naito, Rajavel Elango

**Affiliations:** 1 Research Institute for Bioscience Products and Fine Chemicals, Ajinomoto Co. Inc., Kawasaki 210-8681, Japan; 2 Bio and Fine Chemicals Division, Ajinomoto Co. Inc., Tokyo 104-8351, Japan; 3 Miura Medical Clinic, Osaka 530-0044, Japan; 4 Department of Pediatrics, University of British Columbia, Vancouver, BC V6T 1Z4, Canada; 5 BC Children’s Hospital Research Institute, BC Children’s Hospital, Vancouver, BC V6H 3N1, Canada

**Keywords:** amino acid, NOAEL, randomized controlled clinical trial, supplementation, tyrosine

## Abstract

Background: L-tyrosine, classified as a dispensable amino acid, is widely consumed as a component of commonly consumed foods and as a dietary supplement. However, 4-week safety data on supplementation with this amino acid remain limited. Methods: The aim of this study was to evaluate the safety and tolerability of L-tyrosine supplementation over a 4-week period and to estimate the no-observed-adverse-effect level (NOAEL). In a randomized, double-blind, placebo-controlled crossover trial, 30 healthy adult men received L-tyrosine at graded daily doses (0, 1, 2, 3, or 4 g/day). Each participant received four of the five doses in a randomized sequence, with each intervention period separated by a 2-week washout period. The primary endpoints were clinical laboratory parameters, and the secondary endpoint was the incidence of adverse events. Anthropometric and dietary parameters were also assessed. In addition, plasma amino acid concentrations following L-tyrosine supplementation were evaluated as exploratory outcomes. Results: No clinically meaningful or statistically significant dose-related abnormalities were observed in hematological, biochemical, or electrolyte parameters at any dose. Anthropometric and dietary parameters remained unchanged. No serious adverse events occurred, and the incidence of mild-to-moderate adverse events was comparable to that observed with placebo. At the end of each supplementation period and under fasting conditions, plasma L-tyrosine concentrations modestly increased at the highest dose (4 g/day), whereas concentrations of other amino acids remained unchanged. Conclusions: Four-week supplementation with L-tyrosine at doses up to 4 g/day was well tolerated in healthy adult men and was not associated with biochemical and clinically relevant adverse effects under the conditions of this study. These findings suggest that 4 g/day represents the highest tested intake level without observable adverse effects and may serve as the NOAEL under the present 4-week study conditions.

## 1. Introduction

**Figure 1:**
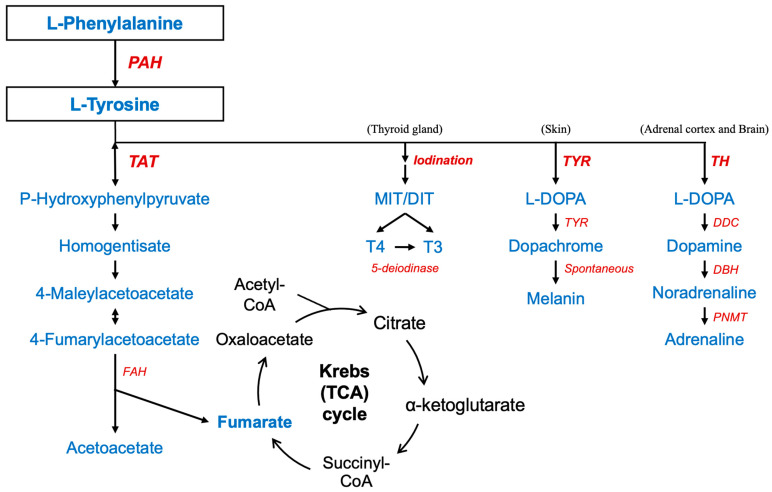
Simplified schematic overview of L-tyrosine catabolism and its major metabolic fates. L-tyrosine is produced from L-phenylalanine by phenylalanine hydroxylase (PAH). L-tyrosine is converted to fumarylacetoacetate and subsequently metabolized to fumarate by fumarylacetoacetate hydrolase (FAH). Fumarate enters the tricarboxylic acid (TCA) cycle, thereby contributing to the generation of reducing equivalents and ATP production via the mitochondrial respiratory chain. In addition, L-tyrosine serves as a precursor for several biologically important molecules. In the adrenal medulla and the central and peripheral nervous systems, L-tyrosine is converted by tyrosine hydroxylase (TH) to L-3,4-dihydroxyphenylalanine (L-DOPA), which is subsequently converted to dopamine by DOPA decarboxylase (DDC). Dopamine is then converted to noradrenaline by dopamine β-hydroxylase (DBH) and subsequently to adrenaline by phenylethanolamine N-methyltransferase (PNMT). In the skin, L-tyrosine is converted by tyrosinase (TYR) via L-DOPA to melanin. In the thyroid gland, tyrosine residues within thyroglobulin are iodinated to form monoiodotyrosine (MIT) and diiodotyrosine (DIT), which are subsequently coupled to generate the thyroid hormones tetraiodothyronine (thyroxine, T4) and triiodothyronine (T3). T4 is further converted to T3 by deiodinases.

L-Tyrosine (L-Tyr) is an aromatic, dispensable amino acid in humans, primarily derived from dietary proteins and from the recycling of endogenous proteins [1]. L-Tyr can also be synthesized primarily in the liver from the indispensable amino acid L-phenylalanine [2]. In addition to serving as a substrate for protein synthesis, L-Tyr is catabolized by tyrosine aminotransferase (TAT) to p-hydroxyphenylpyruvate and subsequently converted to fumarate in several steps, thereby contributing to ATP production via the tricarboxylic acid (TCA) cycle (Figure 1). In addition, acetoacetate generated from L-Tyr can be converted to acetyl- coenzyme A (CoA) in two steps, and acetyl-CoA can then be condensed with oxaloacetate to allow the synthesis of the TCA cycle intermediate citrate. Furthermore, L-Tyr serves as a biosynthetic precursor of melanin in the skin [3,4]. This pigment protects the skin against ultraviolet-induced damage [5]. L-Tyr is also used as a precursor for the synthesis of the hormone thyroxine in the thyroid gland [6,7]. Lastly, L-Tyr is the precursor for the synthesis of catecholamines, including dopamine, noradrenaline (also called norepinephrine) and adrenaline (also called epinephrine), in the adrenal glands and in the central and peripheral nervous systems [8,9,10,11]. Dopamine acts as a neuromodulator [12], while noradrenaline is involved as a neurotransmitter in diverse cognitive processes [13] and as a hormone released from the adrenal medulla [14]. Like noradrenaline, adrenaline acts both as a neurotransmitter and a hormone [15].

L-Tyr is present in commonly consumed foods, including dairy products, soy products, and meat, and plays a critical role as a precursor for the synthesis of biologically active compounds [16,17]. The usual consumption (UC) of L-Tyr in healthy adults has been estimated at a mean of 39.9 mg/kg/day, corresponding to approximately 2.79 g/day for a 70 kg individual [18]. However, the average UC of L-Tyr in Western populations may mask substantial inter-individual variability in the intake of this and other amino acids across different subgroups [19]. Several studies have investigated the physiological and metabolic effects of oral L-Tyr supplementation in humans. Supplementation with 2.5 g/day of L-Tyr for 2 weeks had no significant effect on blood pressure in untreated patients with mild essential hypertension [20]. Administration of 100 mg/kg/day of L-Tyr under acute stress conditions improved several stress-related response markers [21]. Supplementation with 9 g/day of L-Tyr for 4 weeks was not clinically effective in subjects with narcolepsy and cataplexy [22]. In sleep-deprived young men, supplementation with 150 mg/kg/day of L-Tyr improved several performance-related parameters during overnight work [23]. In patients with nemaline myopathy, supplementation with 250–3000 mg/day of L-Tyr was associated with improvements in bulbar function, activity levels, and exercise tolerance [24]. Supplementation with 2 g/day of L-Tyr enhanced cognitive control in healthy young women under specific experimental conditions [25]. Furthermore, a case report described improved muscle strength following L-Tyr supplementation in a neonate with nemaline rod myopathy [26].

Despite these findings, studies evaluating multiple dose levels, medium- to long-term supplementation, and comprehensive clinical safety parameters following oral L-Tyr administration in humans remain limited [27,28]. To the best of our knowledge, no study has systematically determined the no-observed-adverse-effect level (NOAEL) for L-Tyr following 4-week supplementation in humans.

Accordingly, the aim of the present randomized, double-blind, placebo-controlled crossover clinical trial was to evaluate the safety of graded doses of L-Tyr (1, 2, 3, and 4 g/day), administered as dietary supplements for 4 weeks, in healthy adult men compared with placebo.

The primary objective was to identify the NOAEL for L-Tyr supplementation based on clinical laboratory parameters, while treatment-related adverse events were assessed as secondary endpoints.

## 2. Materials and Methods

### 2.1. Study Design

In this randomized, double-blind, placebo-controlled crossover trial, 30 healthy adult men were allocated to five sequence groups according to a randomized crossover design. Within each sequence group, participants received four of the five possible doses (0, 1, 2, 3, or 4 g/day) in a randomized order. Each intervention period generated 24 observations per dose level, and all participants completed the four crossover periods.

### 2.2. Ethical Approval, Clinical Trial Registration and Informed Consent

The clinical trial procedures were conducted in accordance with the Declaration of Helsinki and the Ethical Guidelines for Medical and Biological Research Involving Human Subjects. The study was approved by the Ajinomoto Co., Inc. Human Research Ethics Review Committee (IRB No. 19000012; Study No. 2024-015) on 24 September 2024 and the Medical Corporation Kanonkai Miura Clinic Ethics Review Committee (IRB No. 17000161; Study No. R2402) on 26 September 2024. This study was registered with the Japan Registry of Clinical Trials (jRCT1050240153) on 8 October 2024. Written informed consent was obtained from all participants prior to enrollment.

### 2.3. Randomization and Allocation Concealment

Randomization sequences were generated by an independent researcher using stratified block randomization. To minimize potential time and sequence effects in the crossover phase, dose levels were assigned in a randomized order within each intervention period, consistent with previous studies [29,30]. Allocation was concealed by an independent third party. The principal investigator, study staff, and participants were blinded to the treatment assignments throughout the study.

### 2.4. Washout

A washout period of at least 2 weeks was implemented between intervention periods, consistent with similar protocols used in previous studies [29,30,31,32]. The 2-week washout period was selected based on previous studies evaluating amino acid supplementation and was considered sufficient to minimize potential carryover effects.

### 2.5. Sample Size Determination

As the safety of continuous 4-week L-Tyr supplementation has not previously been evaluated, the sample size was determined based on prior studies assessing the safety of other amino acids using comparable methodologies. These studies indicated that 12–24 participants were sufficient to detect significant changes in plasma amino acid concentrations [29,30,32]. Accordingly, 24 observations per dose level were included in the present study. A post hoc exploratory power analysis for the primary endpoint (creatinine) was conducted using G*Power version 3.1.9.6 [33] for repeated-measures ANOVA (within-factor design). The effect size was derived from the observed partial eta squared (*ηp*^2^ = 0.008; Cohen’s *f* = 0.090), assuming a correlation among repeated measures of 0.5 and a nonsphericity correction *ε* of 1.00. The achieved statistical power was estimated to be 0.696, indicating moderate power for detecting small effect sizes.

### 2.6. Participants

Healthy men aged 20–60 years were recruited in Osaka, Japan, through public advertisement between October 2024 and May 2025. Participants were community-dwelling individuals who maintained their usual daily activities throughout the study period. Screening procedures included a medical history assessment and a physical activity questionnaire, as well as documentation of current and recent medication or supplement use. The inclusion criterion was healthy male adults aged 20–60 years at the time of providing written informed consent.

Exclusion criteria were as follows: (1) regular use or anticipated need for medicinal products during the trial period; (2) use of amino acid-containing supplements from enrollment until study completion; (3) concurrent or recent (within 4 weeks) participation in another clinical trial; (4) history of cardiovascular, hepatic, renal, respiratory, metabolic (including diabetes), or gastrointestinal disorders; (5) presence of any chronic disease requiring medical treatment; (6) known food or drug allergies; and (7) any other condition considered by the study physician to render the participant unsuitable for inclusion.

In addition, individuals with thyroid disorders, including hyperthyroidism and Graves’ disease, and those receiving treatment with monoamine oxidase inhibitors (MAOIs) were excluded from participation in the study.

### 2.7. Experimental Supplemental Food Preparations

Purified amino acids (>99% purity) in the form of L-tyrosine (L-Tyr; Ajinomoto Healthy Supply Co., Inc., Tokyo, Japan) and placebo (corn starch; Japan Corn Starch Co., Ltd., Tokyo, Japan) were encapsulated in size-0 cellulose capsules in accordance with the Japanese Pharmacopoeia.

Daily doses of L-Tyr (0, 1, 2, 3, or 4 g/day) were divided into 10 capsules and provided in coded blister packs. The capsules were prepared and coded by Sumioka Foods Corporation (Shizuoka, Japan). Investigators blinded to group allocation dispensed the supplements to participants.

### 2.8. Intervention

Following baseline assessments, 30 participants were randomized into five groups (n = 6 per group), with stratification to ensure balance in body mass index (BMI), blood pressure, and heart rate. Within each group, participants were assigned to different dose levels (0, 1, 2, 3, or 4 g/day) using stratified block randomization. Each participant received four of the five dose levels for 4 weeks each in a crossover manner. The order of dose administration within each intervention period was randomized to control for potential time-related effects [29,30]. The crossover design reduced the required sample size, minimized inter-individual variability, and enabled a more precise evaluation of clinical laboratory parameters [34]. Participants were instructed to consume the capsules three times daily with their regular meals. Compliance was calculated as the ratio of the number of capsules consumed to the number prescribed, based on participants’ self-reported records.

### 2.9. Withdrawal Criteria

Participants were withdrawn from the study for any of the following reasons: (1) voluntary withdrawal of consent; (2) determination of ineligibility after enrollment; (3) occurrence of adverse events requiring discontinuation, as judged by the study physician; (4) termination of the trial; or (5) a decision by the principal investigator for other justified reasons. No additional participants were recruited after enrollment, even in cases of missing data resulting from withdrawal.

### 2.10. Outcome Measures

#### 2.10.1. Primary Endpoint

At baseline and at the end of each 4-week intervention period, participants visited Miura Medical Clinic between 9:00 and 11:30 a.m. after an overnight fast of at least 8 h. Fasting blood samples were collected for hematological and biochemical analyses. All laboratory analyses were performed by LSI Medience Corporation (Tokyo, Japan) using standardized clinical methods.

Hematological parameters included red blood cell count (RBC), hemoglobin (HGB), hematocrit (HCT), white blood cell count (WBC), and platelet count (PLT). Biochemical parameters included liver enzymes: aspartate aminotransferase (AST), alanine aminotransferase (ALT), γ-glutamyl transferase (γ-GTP), alkaline phosphatase (ALP), and lactate dehydrogenase (LDH); renal function markers: blood urea nitrogen (BUN), creatinine (CRE), and estimated glomerular filtration rate (eGFR); electrolytes: sodium (Na), potassium (K), chloride (Cl), and calcium (Ca); lipid profile: total cholesterol (TC), triglycerides (TG), high-density lipoprotein cholesterol (HDL-C), and low-density lipoprotein cholesterol (LDL-C); proteins: total protein (TP) and albumin (ALB); total bilirubin (T-BIL); creatine kinase (CK); uric acid (UA); glucose (GLU); and phospholipids (PL). The eGFR was calculated in accordance with the 2023 guidelines of the Japanese Society of Nephrology [35].

#### 2.10.2. Secondary Endpoint

The secondary endpoints were the incidence and severity of adverse events (AEs) occurring during the supplementation period. All AEs were recorded from the initiation of the intervention until completion of the supplementation period. Mild AEs were defined as events not requiring treatment; moderate AEs as those requiring short-term, minimal pharmacological intervention; and severe AEs as those requiring hospitalization. The assessment and grading of AEs were conducted in accordance with the Common Terminology Criteria for Adverse Events (CTCAE), version 5.0 [36].

#### 2.10.3. Anthropometric Measurements and Dietary Assessment

Assessments included anthropometric measurements (height, weight, and body mass index [BMI]), as well as blood pressure and heart rate. Height and weight were measured to the nearest 0.1 cm and 0.1 kg, respectively, using an automated height–weight scale (NGP-150L; Nitto Kagaku Co., Ltd., Nagoya, Japan). BMI was calculated as weight (kg) divided by height squared (m^2^). Resting systolic and diastolic blood pressure and heart rate were measured in the seated position using an automated digital monitor (HEM-1000; Omron Healthcare Co., Ltd., Kyoto, Japan). Dietary intake was assessed using the Brief-type Self-Administered Diet History Questionnaire (BDHQ) to estimate daily energy and macronutrient intake [37,38].

### 2.11. Exploratory Measures: Plasma Amino Acids Analysis

Fasting blood samples for amino acid profiling were collected into heparinized tubes and centrifuged at 3000 rpm for 10 min at 4 °C. Plasma was separated and stored at −80 °C until analysis.

In the placebo and L-Tyr groups, plasma concentrations of 19 amino acids, L-histidine (His), L-isoleucine (Ile), L-lysine (Lys), L-methionine (Met), L-phenylalanine (Phe), L-threonine (Thr), L-tryptophan (Trp), L-valine (Val), L-alanine (Ala), L-arginine (Arg), L-aspartic acid (Asp), L-asparagine (Asn), L-cystine (Cys), L-glutamic acid (Glu), L-glutamine (Gln), glycine (Gly), L-proline (Pro), L-serine (Ser), and L-tyrosine (Tyr), were quantified using liquid chromatography–tandem mass spectrometry (LC–MS/MS; Xevo TQ-S micro, Nihon Waters K.K., Tokyo, Japan). All analyses were performed by LSI Medience Corporation (Tokyo, Japan).

### 2.12. Safety Intake Level as NOAEL

The no-observed-adverse-effect level (NOAEL) was defined as the highest experimental intake dose at which no measurable adverse effects were observed in the primary endpoints (hematological and biochemical parameters), secondary endpoints (adverse events [AEs]), or anthropometric and dietary parameters during the 4-week administration period.

### 2.13. Statistical Analysis

Statistical analyses were conducted in both the intention-to-treat (ITT) population, which included all participants with post-enrollment data, and the per-protocol set (PPS), comprising participants who completed all interventions in accordance with the study protocol. Missing data were not imputed. Data are presented as mean ± standard deviation (SD), unless otherwise specified.

Differences in plasma L-Tyr concentrations between baseline and the post-intervention placebo condition were assessed using Student’s t test. Comparisons across different intake levels (0, 1, 2, 3, and 4 g/day) were analyzed using linear mixed-effects models, with dose included as a fixed effect and participant included as a random effect to account for repeated measurements. When a significant main effect of dose was detected, post hoc comparisons were performed using Dunnett’s test to compare each supplementation dose with the placebo condition (0 g/day), with adjustment for multiple comparisons. Categorical variables, including the incidence of adverse events, were analyzed using the chi-square test. Correlations between post-intervention plasma L-Tyr concentrations and related amino acids were evaluated using Pearson’s correlation coefficients (*r*). Linear regression analyses were performed, and corresponding *p* values were calculated. All statistical analyses were conducted using SPSS version 29.0 (IBM Japan, Tokyo, Japan) and Microsoft 365 (version 16; Microsoft Corp., Redmond, WA, USA). All tests were two-sided, and *p* < 0.05 was considered statistically significant.

## 3. Results

### 3.1. Participants

**Figure 2:**
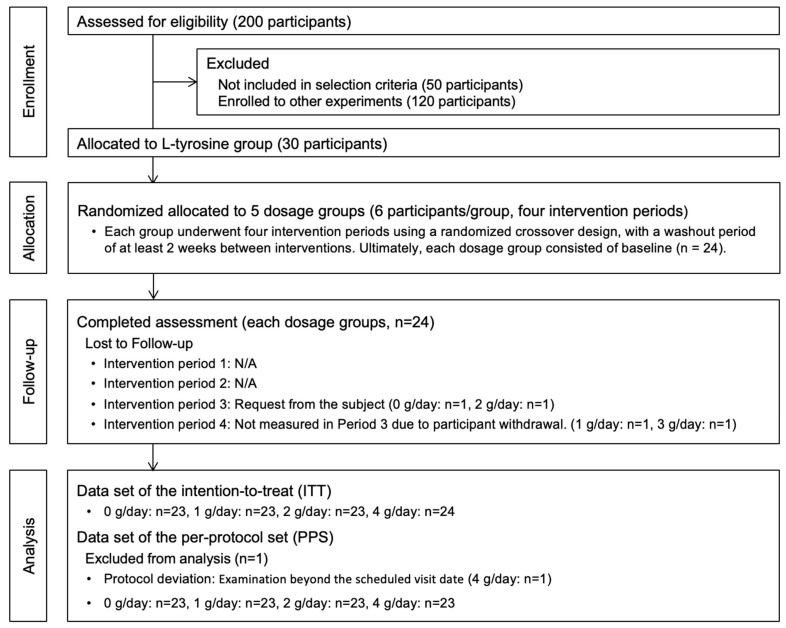
Flow diagram of participants through each stage of the study. The diagram was prepared in accordance with the Consolidated Standards of Reporting Trials (CONSORT) 2025 guidelines [39] to illustrate participant flow throughout the randomized trial.

After written informed consent was obtained, 200 individuals who agreed to participate underwent screening examinations. Following screening and baseline assessments, 30 participants were enrolled by the principal investigator, a physician involved in this study. The flow of participants through each stage of the study is shown in Figure 2, in accordance with the Consolidated Standards of Reporting Trials (CONSORT) 2025 guidelines [39].

A total of 30 participants were allocated to the L-Tyr supplementation study. Baseline characteristics are summarized in Table 1 (plasma amino acid concentrations data are shown in Supplementary Table S1). All 30 participants were included in the intention-to-treat (ITT) population. Participants were randomly assigned to one of five dosage sequences (0 [placebo], 1, 2, 3, and 4 g/day), with six participants per sequence. As described in the Materials and Methods, each participant received each assigned dose for 4 weeks, followed by a washout period of at least 2 weeks. A double-blind, randomized crossover design was employed such that each participant received four of the five dose levels in a randomized order. Accordingly, 24 observations contributed to the analysis for each dose level.

During the intervention period, two participants discontinued the study. The reasons for withdrawal were unrelated to consumption of the test meals. All available data from these participants were included in the ITT analysis, and no replacements were made. In addition, one protocol deviation was identified during the study. The deviation was due to a study visit conducted outside the prespecified allowable visit window defined in the protocol. Data from participants who adhered to the protocol were included in the per-protocol set (PPS) analysis (Figure 2). Compliance with capsule intake was ≥99%.

### 3.2. Primary Endpoints

**Figure 3:**
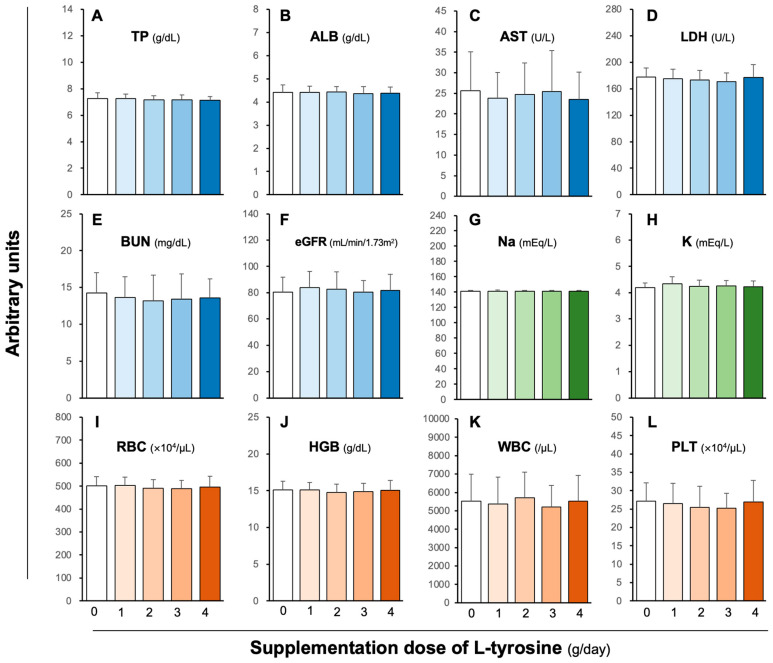
Primary endpoints measured at the end of the supplementation period. Biochemical tests (**A**–**F**): TP, total protein; ALB, albumin; AST, aspartate aminotransferase; LDH, lactate dehydrogenase; BUN, blood urea nitrogen and eGFR, estimated glomerular filtration rate. Serum electrolyte measurements (**G**–**H**): Na, sodium and K, potassium and hematological tests (**I**–**L**): RBC, red blood cells; HGB, hemoglobin; WBC, white blood cells and PLT, platelets. Participants visited the clinic after an overnight fast at the end of each supplementation period for measurements. All values are presented as means ± SD (n = 23, PPS). Statistical significance was assessed using Dunnett’s multiple comparison test versus the placebo group. No statistically significant differences were observed for any parameter compared with the placebo group.

The typical results of hematological parameters, blood biochemical parameters, and serum electrolyte measurements at the end of the L-Tyr supplementation period are presented in Figure 3. The results for all evaluation items, including typical outcomes, are presented in Supplementary Table S2. No statistically significant differences were observed between any L-Tyr supplementation group and the placebo group (0 g/day) for any of the assessed parameters, irrespective of the administered dose (1, 2, 3 or 4 g/day). Moreover, no clinically meaningful changes were observed in any parameter throughout the intervention period. The results of the PPS analysis were consistent with those of the ITT population. As shown in Supplementary Table S2, CK in the 3 g/day group was 280.0 ± 685 U/L, indicating a very large standard deviation. This reflects a marked elevation in CK observed in a single participant (3408 U/L), which contributed to the increased mean and variability. The elevation in CK in this participant was confirmed through consultation with the principal investigator to be transient and attributable to excessive physical exercise. In accordance with the ITT principle, this participant was not excluded from the statistical analysis. However, for reference, the CK values excluding this participant were 137.8 ± 66.4 U/L.

### 3.3. Secondary Endpoints

The secondary endpoints included the incidence and severity of adverse events (AEs) occurring during the supplementation period. Table 2 summarizes the number of AEs observed at each L-Tyr dose level. A total of 18 AEs were reported, of which 9 (50.0%) were classified as mild and 9 (50.0%) as moderate. No serious AEs were observed. Mild AEs included upper respiratory infection, fatigue, bronchitis, pyrexia, and headache, whereas moderate AEs included upper respiratory infection, viral infection, gastrointestinal disorders, apathy, and dermatitis.

The distribution of AEs across the L-Tyr dose groups was compared using the chi-square test. Although a statistically significant difference in AE incidence was detected among the treatment groups (*p* = 0.04), no dose-dependent increase in AE frequency was observed. The apparent difference was likely attributable to the lower AE incidence in the 3 g/day group relative to the other groups.

### 3.4. Anthropometric Measurements and Dietary Intake Estimation

The results of anthropometric measurements (body weight, body mass index, blood pressure, and heart rate) and dietary intake assessments (total energy, protein, fat, and carbohydrate intake) at the end of the supplementation period are summarized in Table 3. No significant differences in anthropometric parameters or dietary intake were observed between any L-Tyr supplementation group and the placebo group at any dose level (1, 2, 3, or 4 g/day). The results obtained in the PPS were consistent with those in the ITT group.

### 3.5. Exploratory Measures: Plasma Amino Acid Analysis

**Figure 4:**
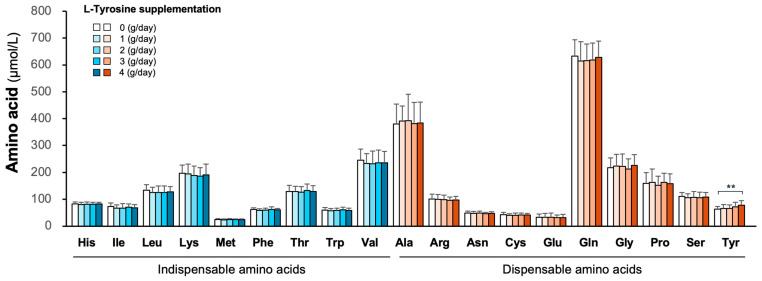
Plasma amino acid concentrations measured at the end of the supplementation period. Participants attended the clinic after an overnight fast at the end of each supplementation period for measurement of plasma amino acid concentrations including: indispensable amino acids (His, L-histidine; Ile, L-isoleucine; Leu, L-leucine; Lys, L-lysine; Met, L-methionine; Phe, L-phenylalanine; Thr, L-threonine; Trp, L-tryptophan and Val, L-valine) and dispensable amino acids (Ala, L-alanine; Arg, L-arginine; Asn, L-asparagine; Cys, L-cystine; Glu, L-glutamate; Gln, L-glutamine; Gly, glycine; Pro, L-proline; Ser, L-serine and Tyr, L-tyrosine). All values are presented as means ± SD (n = 23, PPS). Plasma L-aspartate concentrations were below 5 μmol/L. Statistical significance was assessed using Dunnett’s multiple comparison test versus the placebo group. Statistical significance was defined as ** *p* < 0.01.

The results of plasma amino acid analyses performed after an overnight fast at the end of the supplementation period are summarized in Figure 4 (detailed data are provided in Supplementary Table S3).

In the L-Tyr supplementation groups, plasma L-Tyr concentrations were significantly increased at an intake of 4 g/day (1.23-fold, *p* < 0.01). The concentrations of all other plasma amino acids remained unchanged at all intake levels compared with the placebo group. The results obtained in the PPS were consistent with those observed in the ITT population.

Furthermore, plasma L-Tyr concentrations measured after randomized administration of four dose levels (1, 2, 3, and 4 g/day), each separated by a 2-week washout period, were comparable to baseline values obtained prior to supplementation (63.0 ± 9.8 vs. 60.6 ± 11.4 µmol/L, respectively), with no statistically significant difference (two-tailed Student’s t test, *p* = 0.496). These findings are consistent with previous studies showing that plasma L-Tyr concentrations return to baseline after L-Tyr administration and do not accumulate in the body [40]. Collectively, these results indicate that potential carryover effects in this crossover study were negligible.

## 4. Discussion

### 4.1. Safety Intake Level

The present study provides robust evidence that daily supplementation with L-Tyr at doses up to 4 g/day for 4 weeks is well tolerated in healthy adult men and does not adversely affect metabolic health indices or clinical safety markers. Increasing the dose of L-Tyr up to 4 g/day did not result in significant changes in the primary endpoints (blood parameters) or in secondary observational measures, including the detection of adverse events, anthropometric indices, dietary intake, blood pressure, and heart rate.

No evidence of metabolic dysfunction or impairment of hepatic, renal, muscular, or bone function was observed. Cardiovascular-related markers remained within normal ranges, and no signs of cardiovascular dysfunction were detected. Glycemic parameters, including fasting glucose, and lipid profiles (total cholesterol, HDL-cholesterol, LDL-cholesterol, and triglycerides) were unaffected by 4 weeks of supplementation. In addition, blood electrolyte concentrations and hematological parameters remained stable across all dose groups. Dietary intake, including total energy and macronutrient consumption, was likewise unchanged. Together, these findings indicate that L-Tyr supplementation within the investigated dose range does not appear to adversely influence glucose or lipid metabolism, nor does it compromise systemic metabolic homeostasis.

Although a statistically significant difference in AE incidence was observed among treatment groups (*p* = 0.04), no dose-dependent trend was identified. The difference was primarily attributable to a lower incidence of mild and moderate adverse events in the 3 g/day L-Tyr group when compared to the placebo group rather than a stepwise increase at higher doses. In the absence of a dose–response relationship, this finding is unlikely to represent a treatment-related effect. Accordingly, the frequency and distribution of mild adverse events were comparable between the placebo and L-Tyr groups and showed no dose-related pattern. All reported adverse events resolved during continued supplementation, further supporting the favorable tolerability profile of L-Tyr at doses up to 4 g/day. Consistent with these observations, the absence of changes in dietary intake throughout the intervention suggests that L-Tyr supplementation does not affect appetite regulation or habitual macronutrient consumption, thereby minimizing the potential for indirect metabolic effects mediated by alterations in energy intake.

Taken together, these results support a NOAEL for L-Tyr of 4 g/day (57.9 mg/kg/day) in healthy adult men under the present 4-week study conditions. This NOAEL corresponds to the highest dose evaluated in the present study and aligns with dosing regimens previously employed within intake ranges considered safe in clinical investigations [29,30,32,41,42,43]. In a prior study by Elwes et al., L-Tyr was administered at a higher dose (9 g/day) for 4 weeks in patients with narcolepsy and cataplexy; however, comprehensive safety evaluations were not conducted in this study. Thus, the present study provides clinical, biochemical, anthropometric, and dietary evidence that substantiates the safety margin of 4-week L-Tyr supplementation in healthy adult men.

Stepwise dose-escalation trials have established NOAELs for indispensable amino acids, including L-methionine, L-histidine, L-phenylalanine, L-threonine, and L-tryptophan; dispensable amino acids such as L-arginine, glycine, and L-serine; and non-proteinogenic amino acids including L-ornithine and L-citrulline. Collectively, these studies indicate that relatively high doses of single amino acids do not substantially disrupt metabolic homeostasis or impair major organ function during short- to 4-week intake in healthy adult men. However, it is worth noting that the proposed NOAEL values differ between the amino acids tested in supplements, thus indicating variable tolerability according to the amino acid tested.

Accordingly, the present study extends the established framework of amino acid tolerability research to L-Tyr and addresses a clinically relevant gap in the literature. To the best of our knowledge, this is the first study to report a NOAEL for L-Tyr following 4-week supplementation in humans under the present study conditions.

### 4.2. Plasma Amino Acids

**Figure 5:**
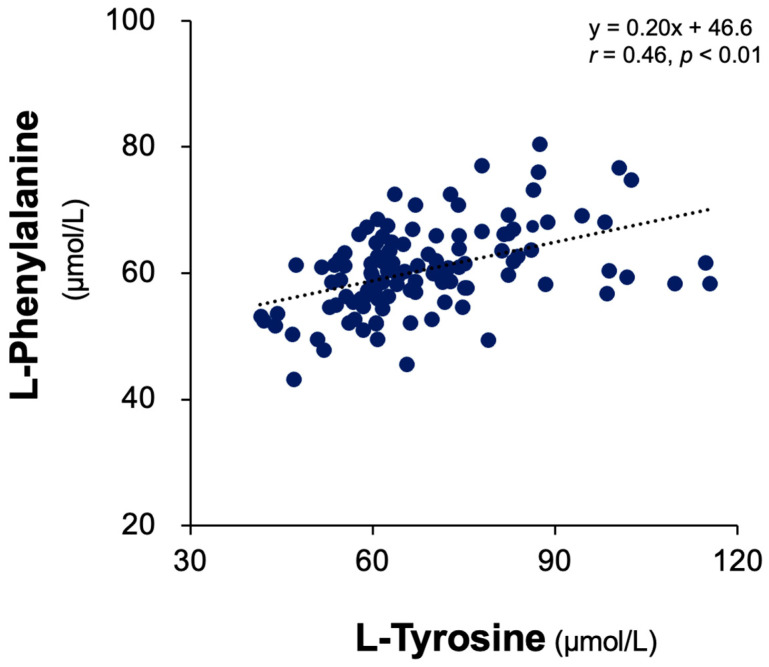
Correlation between post-intervention plasma L-tyrosine and L-phenylalanine concentrations. Linear regression analysis was performed, and Pearson’s correlation coefficient (r) and the corresponding *p* value are shown (n = 115, PPS).

In the present study, we investigated the effects of L-tyrosine (L-Tyr) supplementation on plasma amino acid profiles and related metabolic pathways. Plasma L-Tyr concentrations were modestly but significantly elevated in the highest-dose group (4 g/day) compared with the placebo group. However, no dose-dependent changes were observed in the concentrations of other amino acids, including its precursor L-phenylalanine (L-Phe) (Figure 1). Furthermore, as described above, clinical and biochemical assessments revealed no findings suggestive of metabolic disturbances or organ dysfunction (Table 2). Because the digestion of dietary and endogenous proteins and the absorption of the resulting amino acids are largely completed after an overnight fast [44,45], the plasma L-Tyr profile observed after 4 weeks of supplementation at the highest dose likely reflects tissue-level adaptations. Although elucidating the potential adaptive mechanisms underlying the observed changes in circulating L-Tyr concentrations is beyond the scope of the present study, several considerations merit discussion. Plasma L-Tyr concentrations represent the net balance between tissue uptake and release. Therefore, the elevated circulating L-Tyr levels observed after 4 weeks of supplementation may result from decreased tissue uptake and/or increased release of L-Tyr. Further studies are required to clarify these mechanisms and to elucidate the biological basis of the present findings. One important aspect of L-Tyr metabolism is that L-Tyr is catabolized by tyrosine aminotransferase (TAT) to p-hydroxyphenylpyruvate and subsequently converted to fumarate, which enters the tricarboxylic acid (TCA) cycle. Through this pathway, L-Tyr contributes to ATP production and serves as an important intermediate in central carbon metabolism. Therefore, increased L-Tyr intake could have theoretically influenced central carbon metabolism. In addition, L-Tyr functions as a biosynthetic precursor of catecholamines [8,9,10,11] (Figure 1), suggesting potential effects on physiological parameters such as blood pressure and glucose metabolism. Moreover, thyroxine (T4), a thyroid hormone synthesized from L-Tyr, acts on virtually all tissues to regulate basal metabolic processes, including carbohydrate, lipid, and protein metabolism, as well as heart rate [6]. However, under the conditions of the present study, supplementation with up to 4 g/day of L-Tyr did not produce measurable changes in physiological markers, including blood pressure and glucose metabolism (Supplementary Table S2). Furthermore, aside from the increase in circulating L-Tyr itself, plasma concentrations of other amino acids remained stable (Figure 4 and Supplementary Table S3). A significant correlation (*r* = 0.46, *p* < 0.01) was observed between post-intervention plasma L-Tyr concentrations and those of its precursor, L-Phe (Figure 5). This finding suggests that L-Tyr supplementation may influence L-Phe metabolism in a still unknown manner. Further investigation under conditions exceeding the intake range examined in the present study is warranted to document this new aspect.

### 4.3. Limitations

This study has several limitations. This study did not evaluate doses exceeding 4 g/day, longer-term intake, or populations including women and individuals with underlying medical conditions.

First, the clinical trial included only healthy participants aged 20–60 years. This is consistent with the recommendation that, for safety reasons, the NOAEL values for amino acid supplements should first be established in healthy adults before being evaluated in specific subpopulations for whom amino acid supplementation may be beneficial [19].

Second, this trial included only male participants. Previous rodent studies have suggested that NOAEL values for certain amino acids may differ between males and females [27]. For L-Tyr supplementation, different NOAEL values have been reported for male and female rats [28]. Although animal models provide useful preliminary data, direct extrapolation from rodents to humans is not possible. Thus, clinical trials including female participants are necessary to determine whether the NOAEL identified in men in the present study is also applicable to women.

Third, the 4-week supplementation period was selected based on previous clinical studies [29,30,32,41,42,43] and was considered appropriate, particularly for practical reasons, to assess the NOAEL for L-Tyr. Although the present study provides 4-week safety data, further investigations with longer follow-up periods are warranted.

Fourth, the highest dose evaluated in this study was 4 g/day, and the safety of higher intake levels was not assessed.

Fifth, this study did not assess biomarkers directly related to L-tyrosine metabolism, such as thyroid-stimulating hormone (TSH), triiodothyronine (T3), thyroxine (T4), dopamine, noradrenaline and adrenaline. Therefore, the potential effects of L-tyrosine supplementation on thyroid function and catecholamine-related responses were not directly evaluated. Although no concerns regarding sleep, anxiety, or other mood-related symptoms were identified during physician interviews conducted at each study visit, potential effects of L-tyrosine metabolites on thyroid function, catecholamine-related responses, and other neuropsychological outcomes cannot be completely ruled out.

Lastly, although the Brief-type Self-administered Diet History Questionnaire (BDHQ) [37,38] is a validated dietary assessment tool widely used in Japanese populations, it is subject to systematic biases inherent to food frequency questionnaires (FFQs). As a self-administered instrument with predefined food items and standardized portion sizes, the BDHQ is susceptible to recall-related inaccuracies and may not fully capture individual dietary variability, potentially resulting in systematic under- or overestimation of nutrient intake. Although the BDHQ does not provide estimates of individual amino acid intake, the average dietary tyrosine intake among Japanese men aged 30–64 years has been reported to be approximately 3.5 g/day [46]. However, data on individual tyrosine intake were not available because the BDHQ does not allow estimation of specific amino acid intake.

## 5. Conclusions

Under the conditions of the present study, our data collectively indicate that daily supplementation with L-Tyr at doses of up to 4 g/day (equivalent to 57.9 mg/kg/day) for 4 weeks is well tolerated and is not associated with adverse effects, biochemical or hematological abnormalities, or changes in anthropometric and dietary parameters. These findings indicate that this intake level can be considered the NOAEL value for healthy adult men under the conditions of the present study.

## Figures and Tables

**Table 1:** Baseline characteristics of participants.

Parameters	Units	Mean	±	SD
(A) Anthropometric measurements and blood pressure				
Age	year	43.4	±	11.0
Height	cm	172.4	±	6.4
Weight	kg	69.1	±	8.2
Body mass index (BMI)	kg/m^2^	23.2	±	2.2
Systolic blood pressure (SBP)	mmHg	126.7	±	11.9
Diastolic blood pressure (DBP)	mmHg	80.1	±	11.7
Heart rate	bpm	69.9	±	10.1
(B) Dietary assessment				
Total energy	kcal/day	1854	±	517
Fat	g/day	63.1	±	18.1
Protein	g/day	53.6	±	19.3
Carbohydrates	g/day	263.8	±	91.5
(C) Biochemical tests				
TP	g/dL	7.4	±	0.4
ALB	g/dL	4.6	±	0.3
T-BIL	mg/dL	0.89	±	0.30
AST	U/L	23.6	±	8.5
ALT	U/L	22.2	±	11.8
LDH	U/L	173.7	±	26.1
ALP	U/L	75.3	±	16.7
γ-GTP	U/L	24.4	±	11.8
CK	U/L	146.2	±	99.7
PL	mg/dL	226.8	±	25.4
BUN	mg/dL	12.8	±	3.0
CRE	mg/dL	0.847	±	0.09
UA	mg/dL	6.0	±	0.9
eGFR	mL/min/1.73 m^2^	81.0	±	11.9
GLU	mg/dL	88.3	±	6.5
HbA1c	%	5.4	±	0.3
TC	mg/dL	203.7	±	23.3
TG	mg/dL	119.5	±	105.6
HDL-C	mg/dL	59.7	±	14.9
LDL-C	mg/dL	118.9	±	23.7
(D) Serum electrolyte measurements				
Na	mEq/L	141.3	±	1.7
K	mEq/L	4.2	±	0.3
Cl	mEq/L	104.0	±	1.9
Ca	mg/dL	9.4	±	0.3
(E) Hematological tests				
RBC	×10^4^/µL	498.2	±	43.4
HGB	g/dL	15.1	±	1.13
HCT	%	46.9	±	3.5
WBC	/µL	5270	±	1395
PLT	×10^4^/µL	26.4	±	5.1

Baseline characteristics of Groups A–E are shown. All values are presented as means ± SD (n = 30, pre-intervention participants). Participants visited the clinic in an overnight fast state prior to supplementation for baseline measurements. (A) Anthropometric assessments (weight; BMI, body mass index), blood pressure (SBP, systolic blood pressure; DBP, diastolic blood pressure), heart rate measurements. (B) Dietary assessments (total energy; protein; fat; carbohydrate). (C) Biochemical tests (TP, total protein; ALB, albumin; T-BIL, total bilirubin; AST, aspartate aminotransferase; ALT, alanine aminotransferase; LDH, lactate dehydrogenase; ALP, alkaline phosphatase; γ-GTP, gamma-glutamyl transferase; CK, creatine kinase; PL, phospholipids; BUN, blood urea nitrogen; CRE, creatinine; UA, uric acid; eGFR, estimated glomerular filtration rate; GLU, glucose; TC, total cholesterol; TG, triglycerides; HDL-C, high-density lipoprotein cholesterol; LDL-C, low-density lipoprotein cholesterol). (D) Serum electrolyte measurements (Na, sodium; K, potassium; Cl, chloride; Ca, calcium). (E) Hematological tests (RBC, red blood cells; HGB, hemoglobin; HCT, hematocrit; WBC, white blood cells; PLT, platelets). These measurements were conducted before supplementation.

**Table 2:** Secondary endpoints: incidence of adverse events among participants during the supplementation period.

	Supplementation Doses of L-Tyr					Total Counts	
(g/day)	0	1	2	3	4		(%)
Mild	2	2	5	0	0	9	(50.0)
Moderate	2	2	0	1	4	9	(50.0)
Serious	0	0	0	0	0	0	(0.0)
Total	4	4	5	1	4	18	
(%)	(22.2)	(22.2)	(27.8)	(5.6)	(22.2)	(100.0)	

All values represent the number of adverse events (AEs) that occurred during the supplementation period among participants receiving placebo (0 g/day) or L-Tyr at different dose levels. AE severity was classified as follows: mild, minor symptoms that allowed the participant to continue the study without treatment or medication; moderate, symptoms that allowed the participant to continue the study with treatment or medication; and severe, symptoms requiring hospitalization. Differences in the distribution of categorical data (number of AEs) among treatment groups were analyzed using the chi-square test. A statistically significant difference in AE incidence was observed among the treatment groups (*p* = 0.04); however, no dose-dependent trend was identified.

**Table 3:** Anthropometric measurements, blood pressure, and dietary assessment at the end of the supplementation period.

Parameters	Units	Supplementation Doses of L-Tyr				
		0 g/day	1 g/day	2 g/day	3 g/day	4 g/day
(A) Anthropometric measurements and blood pressure						
Weight	kg	71.3 ± 8.6	70.3 ± 8.4	70.5 ± 9.2	70.2 ± 8.3	69.3 ± 8.3
BMI	kg/m^2^	23.5 ± 2.4	23.6 ± 2.3	23.6 ± 2.2	23.9 ± 2.3	23.5 ± 2.4
SBP	mmHg	124.3 ± 15.6	127.1 ± 16.0	126.4 ± 16.3	126.6 ± 16.8	125.2 ± 12.1
DBP	mmHg	79.8 ± 14.1	82.0 ± 14.5	79.9 ± 14.6	80.5 ± 13.2	79.1 ± 9.4
Heart rate	bpm	69.5 ± 8.0	73.1 ± 11.1	72.7 ± 11.3	72.2 ± 12.2	72.3 ± 10.0
(B) Dietary assessment						
Total energy	kcal/day	1708 ± 480	1671 ± 581	1666 ± 446	1851 ± 445	1772 ± 529
Protein	g/day	61.2 ± 17.5	59.4 ± 19.3	57.1 ± 16.9	62.3 ± 15.4	60.6 ± 19.7
Fat	g/day	48.8 ± 14.8	47.4 ± 16.3	45.5 ± 15.2	51.3 ± 14.5	49.7 ± 15.8
Carbohydrates	g/day	239.7 ± 80	230.6 ± 93	235.6 ± 71	260.3 ± 81	251.0 ± 91

Participants attended the clinic after an overnight fast at the end of individual L-Tyr supplementation period for anthropometric (weight; BMI, body mass index), blood pressure (SBP, systolic blood pressure; DBP, diastolic blood pressure), heart rate measurements and dietary assessments (total energy; protein; fat; carbohydrate). All values are presented as means ± SD (n = 23, PPS). Statistical significance was assessed using Dunnett’s multiple comparison test versus the placebo group. No statistically significant differences were observed compared with the placebo group.

## Data Availability

All relevant data are included in this manuscript. The data are presented in a format that does not contain any personal or identifiable information.

## Supplementary Materials

The following supporting information can be downloaded at: https://www.mdpi.com/article/10.3390/nu18122020/s1; Supplementary Table S1. Baseline plasma amino acid concentrations. Supplementary Table S2. Primary endpoints measured at the end of the supplementation period. Supplementary Table S3. Plasma amino acid concentrations at the end of the supplementation period.
