# Supplementary material for: Evaluation of the Safety and Tolerability of L-Tyrosine Supplementation in Healthy Adult Men: A Randomized Crossover Trial
Source: Nutrients. 2026 Jun 21;18(12):2020. doi: 10.3390/nu18122020 (PMC13304972; doi:10.3390/nu18122020)
Supplement: Supplementary file 1 [file nutrients-18-02020-s001.zip › nutrients-4366974-supplementary.pdf]

Supplementary Table S1. Baseline Plasma Amino Acid Concentrations.

Amino acids	unit	means	±	SD
**Indispensable amino acids**				
His	μmol/L	81.8	±	9.0
Ile	μmol/L	69.0	±	15.6
Leu	μmol/L	123.8	±	25.2
Lys	μmol/L	190.0	±	31.3
Met	μmol/L	23.4	±	3.5
Phe	μmol/L	58.7	±	7.5
Thr	μmol/L	130.2	±	24.9
Trp	μmol/L	60.1	±	8.2
Val	μmol/L	232.5	±	43.3
**Dispensable amino acids**				
Ala	μmol/L	390.4	±	68.7
Arg	μmol/L	97.0	±	15.2
Asp	μmol/L	< 5.0		
Asn	μmol/L	48.0	±	7.8
Cystine	μmol/L	43.2	±	7.7
Glu	μmol/L	32.8	±	14.6
Gln	μmol/L	627.7	±	69.4
Gly	μmol/L	231.5	±	42.3
Pro	μmol/L	158.3	±	30.2
Ser	μmol/L	109.3	±	17.1
Tyr	μmol/L	60.6	±	11.4

Baseline plasma concentrations of indispensable amino acids (His, L-histidine; Ile, L-isoleucine; Leu, L-leucine; Lys, L-lysine; Met, L-methionine; Phe, L-phenylalanine; Thr, L-threonine; Trp, L-tryptophan; Val, L-valine) and dispensable amino acids (Ala, L-alanine; Arg, L-arginine; Asp, L-aspartate; Asn, L-asparagine; Cystine, L-cystine; Glu, L-glutamate; Gln, L-glutamine; Gly, glycine; Pro, L-proline; Ser, L-serine; Tyr, L-tyrosine) are shown. All values are presented as means ± SD (n = 30, pre-intervention participants).

Supplementary Table S2. Primary Endpoints Measured at the End of the Supplementation Period.

Parameter	g/day	means	±	SD	change	*P*
**A) Biochemical tests**						
TP	0	7.25	±	0.43		
(g/dL)	1	7.25	±	0.37	0.00	1.000
RV 6.7-8.3	2	7.17	±	0.30	-0.09	0.829
	3	7.16	±	0.39	-0.09	0.804
	4	7.15	±	0.28	-0.10	0.724
ALB	0	4.41	±	0.32		
(g/dL)	1	4.41	±	0.27	0.00	1.000
RV 3.8-5.2	2	4.44	±	0.22	0.03	0.980
	3	4.37	±	0.29	-0.04	0.970
	4	4.38	±	0.27	-0.03	0.993
T-BIL	0	0.89	±	0.27		
(mg/dL)	1	0.93	±	0.31	0.03	0.982
RV 0.2-1.2	2	0.92	±	0.31	0.03	0.994
	3	0.77	±	0.26	-0.12	0.402
	4	0.85	±	0.26	-0.04	0.972
AST	0	25.6	±	9.5		
(U/L)	1	23.8	±	6.2	-1.8	0.871
RV 10-40	2	24.7	±	7.6	-0.9	0.989
	3	25.4	±	10.0	-0.2	1.000
	4	23.6	±	6.6	-2.0	0.809
ALT	0	25.2	±	14.3		
(U/L)	1	25.5	±	14.5	0.3	1.000
RV 5-45	2	24.7	±	14.1	-0.5	1.000
	3	24.8	±	13.2	-0.4	1.000
	4	25.7	±	18.9	0.5	1.000
LDH	0	177.5	±	26.6		
(U/L)	1	175.3	±	27.8	-2.2	0.996
RV 120-240	2	173.6	±	32.9	-4.0	0.968
	3	170.7	±	25.6	-6.8	0.818
	4	177.4	±	24.7	-0.1	1.000
ALP	0	78.7	±	17.7		
(U/L)	1	77.0	±	13.5	-1.8	0.990
RV 38-113	2	73.4	±	16.2	-5.3	0.665
	3	77.8	±	21.9	-0.9	0.999
	4	75.9	±	14.6	-2.8	0.947
γ-GTP	0	24.7	±	12.9		
(U/L)	1	27.5	±	15.6	2.8	0.950
RV <80	2	28.0	±	15.8	3.3	0.915
	3	28.7	±	25.7	4.0	0.847
	4	24.4	±	11.5	-0.3	1.000
CK	0	156.6	±	68		
(U/L)	1	132.0	±	34	-25	0.997

RV 60-270	2	162.0	±	87	5	1.000
	3	280.0	±	685	123	0.478
	4	152.1	±	102	-4	1.000
PL	0	220.4	±	28.5		
(mg/dL)	1	226.8	±	26.8	6.3	0.884
RV 150-280	2	226.0	±	28.5	5.6	0.923
	3	224.3	±	40.8	3.9	0.978
	4	222.6	±	22.3	2.1	0.998
BUN	0	14.3	±	2.8		
(mg/dL)	1	13.6	±	2.8	-0.6	0.895
RV 8.0-20	2	13.2	±	3.5	-1.1	0.580
	3	13.4	±	3.4	-0.9	0.734
	4	13.6	±	2.6	-0.7	0.850
CRE	0	0.850	±	0.09		
(mg/dL)	1	0.826	±	0.10	-0.02	0.795
RV 0.61-1.04	2	0.835	±	0.10	-0.02	0.949
	3	0.846	±	0.07	0.00	1.000
	4	0.843	±	0.10	-0.01	0.996
UA	0	5.88	±	0.8		
(mg/dL)	1	5.78	±	0.9	-0.1	0.985
RV 3.8-7.0	2	5.82	±	0.8	-0.1	0.998
	3	5.90	±	0.9	0.0	1.000
	4	5.68	±	0.9	-0.2	0.851
eGFR	0	80.5	±	11.2		
(mL/min/1.73m^2)	1	83.8	±	12.4	-3.4	0.732
RV ≥60	2	82.8	±	13.3	-2.3	0.912
	3	80.6	±	8.8	-0.1	1.000
	4	81.6	±	12.5	-1.1	0.993
GLU	0	85.7	±	10.5		
(mg/dL)	1	88.1	±	6.1	2.4	0.711
RV 70-109	2	87.1	±	5.8	1.3	0.946
	3	88.3	±	9.5	2.5	0.673
	4	86.9	±	7.5	1.2	0.967
TC	0	200.9	±	20.1		
(mg/dL)	1	205.5	±	24.9	4.6	0.932
RV 120-219	2	201.6	±	24.9	0.7	1.000
	3	206.5	±	32.1	5.6	0.873
	4	208.9	±	25.2	8.0	0.674
TG	0	116.2	±	130.2		
(mg/dL)	1	109.0	±	74.9	-7.2	0.996
RV 30-149	2	124.6	±	75.8	8.3	0.993
	3	121.6	±	90.9	5.3	0.999
	4	95.6	±	48.7	-20.7	0.844
HDL-C	0	57.6	±	14.1		
(mg/dL)	1	62.4	±	14.2	4.7	0.609
RV 40-85	2	60.4	±	14.9	2.8	0.905
	3	57.4	±	14.3	-0.3	1.000

	4	60.0	±	12.7	2.3	0.946
LDL-C	0	119.6	±	18.7		
(mg/dL)	1	121.8	±	24.5	2.3	0.991
RV 65-139	2	118.7	±	20.6	-0.9	1.000
	3	122.9	±	25.4	3.3	0.964
	4	128.6	±	23.4	9.0	0.464
B) Serum electrolyte measurements						
Na	0	140.8	±	1.4		
(mEq/L)	1	141.1	±	1.5	0.3	0.914
RV 137-147	2	141.0	±	1.2	0.2	0.979
	3	140.9	±	1.3	0.1	0.998
	4	140.9	±	1.3	0.1	0.998
K	0	4.20	±	0.17		
(mEq/L)	1	4.34	±	0.27	0.1	0.142
RV 3.5-5.0	2	4.24	±	0.24	0.0	0.959
	3	4.26	±	0.21	0.1	0.811
	4	4.22	±	0.23	0.0	0.993
Cl	0	103.0	±	1.7		
(mEq/L)	1	103.7	±	1.9	0.7	0.452
RV 98-108	2	103.5	±	1.5	0.5	0.786
	3	103.4	±	2.1	0.4	0.836
	4	103.0	±	1.8	0.0	1.000
Ca	0	9.33	±	0.31		
(mg/dL)	1	9.44	±	0.26	0.1	0.539
RV 8.4-10.4	2	9.39	±	0.30	0.1	0.884
	3	9.34	±	0.32	0.0	1.000
	4	9.32	±	0.35	0.0	1.000
**C) Hematological tests**						
RBC	0	501.0	±	40.7		
($\times 10^4/\mu L$)	1	504.0	±	36.3	3.0	0.997
RV 430-570	2	489.9	±	39.0	-11.1	0.749
	3	489.3	±	35.0	-11.7	0.714
	4	496.9	±	47.0	-4.1	0.990
HGB	0	15.1	±	1.2		
(g/dL)	1	15.1	±	1.0	0.0	1.000
RV 13.5-17.5	2	14.8	±	1.1	-0.3	0.755
	3	14.9	±	1.1	-0.2	0.900
	4	15.0	±	1.4	-0.1	0.997
HCT	0	46.1	±	3.5		
(%)	1	46.4	±	3.1	0.2	0.998
RV 39.7–52.4	2	45.4	±	3.5	-0.8	0.887
	3	45.5	±	3.4	-0.6	0.940
	4	45.9	±	4.3	-0.2	0.999
WBC	0	5539	±	1455		
(/μL)	1	5370	±	1472	-170	0.982
RV 3300-9000	2	5700	±	1397	161	0.985
	3	5204	±	1187	-335	0.829

	4	5522	±	1401	-17	1.000
PLT	0	27.2	±	5.0		
(×10^4/μL)	1	26.5	±	5.5	-0.7	0.972
RV 14-34	2	25.5	±	5.7	-1.7	0.657
	3	25.2	±	4.1	-1.9	0.532
	4	27.0	±	5.8	-0.2	1.000

Participants attended the clinic after an overnight fast at the end of each supplementation period. Biochemical tests, serum electrolyte measurements, and hematological tests are shown. All values are presented as means ± SD (n = 23, PPS). A)Biochemical tests (TP, total protein; ALB, albumin; T-BIL, total bilirubin; AST, aspartate aminotransferase; ALT, alanine aminotransferase; LDH, lactate dehydrogenase; ALP, alkaline phosphatase; γ-GTP, gamma-glutamyl transferase; CK, creatine kinase; PL, phospholipids; BUN, blood urea nitrogen; CRE, creatinine; UA, uric acid; eGFR, estimated glomerular filtration rate; GLU, glucose; TC, total cholesterol; TG, triglycerides; HDL-C, high-density lipoprotein cholesterol; LDL-C, low-density lipoprotein cholesterol). B) Serum electrolyte measurements (Na, sodium; K, potassium; Cl, chloride; Ca, calcium). C) Hematological tests (RBC, red blood cells; HGB, hemoglobin; HCT, hematocrit; WBC, white blood cells; PLT, platelets). Statistical significance was assessed using Dunnett's multiple comparison test versus placebo group. No statistically significant differences were observed versus the placebo group. Reference values (RV) indicate clinical normative values used at Miura Medical Clinic.

Supplementary Table S3. Plasma Amino Acid Concentrations at the End of the Supplementation Period.

Parameter	g/day	means	±	SD	change	*P*
**A) Indispensable amino acid**						
His	0	82.4	±	7.7		
(µmol/L)	1	80.1	±	8.1	-2.3	0.679
RV 68.0-116.6	2	81.7	±	7.8	-0.7	0.993
	3	81.8	±	7.4	-0.6	0.995
	4	82.6	±	6.2	0.2	1.000
Ile	0	72.8	±	14.0		
(µmol/L)	1	67.4	±	11.9	-5.4	0.469
RV 44.9-120.3	2	66.4	±	17.0	-6.4	0.318
	3	70.3	±	12.8	-2.5	0.927
	4	68.5	±	11.6	-4.3	0.665
Leu	0	133.3	±	20.7		
(µmol/L)	1	124.7	±	19.5	-8.6	0.484
RV 84.4-200.2	2	125.1	±	24.8	-8.2	0.522
	3	125.2	±	24.4	-8.1	0.536
	4	127.8	±	19.7	-5.4	0.816
Lys	0	197.3	±	29.5		
(µmol/L)	1	193.9	±	36.8	-3.4	0.992
RV 138.6-294.2	2	188.3	±	35.0	-9.0	0.791
	3	186.5	±	30.4	-10.8	0.670
	4	190.4	±	40.3	-6.9	0.903
Met	0	24.8	±	2.8		
(µmol/L)	1	23.6	±	3.1	-1.2	0.483
RV 18.1-43.5	2	24.4	±	3.9	-0.4	0.973
	3	23.9	±	2.5	-0.9	0.755
	4	24.1	±	3.1	-0.7	0.847
Phe	0	61.9	±	5.7		
(µmol/L)	1	58.6	±	6.0	-3.2	0.314
RV 49.0-90.8	2	59.9	±	6.7	-1.9	0.741
	3	62.0	±	9.5	0.1	1.000
	4	60.8	±	5.2	-1.1	0.955
Thr	0	129.2	±	22.3		
(µmol/L)	1	128.9	±	18.9	-0.2	1.000
RV 89.2-241.6	2	126.6	±	19.9	-2.6	0.983
	3	132.8	±	24.0	3.6	0.943
	4	128.7	±	22.0	-0.4	1.000
Trp	0	59.7	±	9.6		
(µmol/L)	1	57.3	±	8.6	-2.4	0.785
RV 46.7-92.0	2	58.1	±	8.8	-1.6	0.931
	3	60.3	±	10.3	0.6	0.998
	4	58.4	±	8.6	-1.3	0.968
Val	0	245.6	±	41		

(μmol/L)	1	233.7	±	37	-11.8	0.757
RV 162.9-351.4	2	232.0	±	47	-13.5	0.664
	3	236.2	±	47	-9.3	0.875
	4	236.1	±	42	-9.4	0.870
**B) Dispensable amino acids**						
Ala	0	379.9	±	74.5		
(μmol/L)	1	391.6	±	56.0	11.7	0.963
RV 253.6-601.9	2	392.5	±	98.5	12.6	0.952
	3	382.0	±	79.0	2.1	1.000
	4	384.3	±	77.0	4.4	0.999
Arg	0	100.5	±	18.9		
(μmol/L)	1	99.8	±	17.9	-0.7	1.000
RV 44.1-115.2	2	98.8	±	16.5	-1.7	0.990
	3	95.9	±	12.1	-4.5	0.737
	4	97.4	±	13.6	-3.1	0.913
Asn	0	48.5	±	7.4		
(μmol/L)	1	47.4	±	7.8	-1.1	0.955
RV 37.7-78.5	2	48.1	±	7.4	-0.5	0.998
	3	46.2	±	5.1	-2.3	0.614
	4	47.9	±	5.7	-0.6	0.994
Cystine	0	42.6	±	8.1		
(μmol/L)	1	40.4	±	6.4	-2.2	0.689
RV 34.9-77.7	2	40.2	±	8.0	-2.4	0.621
	3	41.8	±	6.8	-0.8	0.989
	4	41.0	±	6.9	-1.6	0.859
Glu	0	32.3	±	12.6		
(μmol/L)	1	32.3	±	15.5	0.0	1.000
RV 13.3-86.7	2	32.4	±	16.5	0.1	1.000
	3	31.9	±	10.0	-0.5	1.000
	4	31.2	±	12.0	-1.1	0.996
Gln	0	632.6	±	61.5		
(μmol/L)	1	614.8	±	72.1	-17.8	0.751
RV 503.4-851.4	2	615.9	±	62.2	-16.7	0.790
	3	617.8	±	63.5	-14.9	0.848
	4	628.3	±	60.7	-4.4	0.998
Gly	0	217.3	±	36.7		
(μmol/L)	1	223.3	±	43.9	6.1	0.965
RV 136.8-397.7	2	222.3	±	46.3	5.1	0.981
	3	212.1	±	38.5	-5.2	0.980
	4	226.5	±	40.0	9.3	0.860
Pro	0	159.5	±	40.0		
(μmol/L)	1	163.4	±	49.2	3.8	0.992
RV 89.8-304.7	2	152.0	±	34.0	-7.5	0.911
	3	163.4	±	33.2	3.9	0.992
	4	158.4	±	35.4	-1.1	1.000
Ser	0	110.4	±	15.3		

(μmol/L)	1	105.8	± 14.9	-4.5	0.815
RV 78.4-200.1	2	107.0	± 22.0	-3.4	0.920
	3	105.2	± 21.6	-5.2	0.736
	4	108.5	± 16.2	-1.8	0.991
Tyr	0	63.0	± 9.8		
(μmol/L)	1	65.7	± 15.0	2.7	0.924
RV 46.7-103.6	2	66.1	± 12.4	3.1	0.882
	3	72.0	± 16.7	8.9	0.126
	4	77.6	± 17.6	14.5	0.004**

Participants attended the clinic after an overnight fast for measurement of plasma amino acid concentrations. A) Indispensable amino acids (His, L-histidine; Ile, L-isoleucine; Leu, L-leucine; Lys, L-lysine; Met, L-methionine; Phe, L-phenylalanine; Thr, L-threonine; Trp, L-tryptophan; Val, L-valine). B) Dispensable amino acids (Ala, L-alanine; Arg, L-arginine; Asn, L-asparagine; Cystine, L-cystine; Glu, L-glutamate; Gln, L-glutamine; Gly, glycine; Pro, L-proline; Ser, L-serine; Tyr, L-tyrosine). All values are presented as means ± SD (n = 23, PPS). Plasma L-aspartate concentrations were below 5 μmol/L. Statistical significance was assessed using Dunnett's multiple comparison test versus the placebo group. Statistical significance was defined as: *$P < 0.05$ and **$P < 0.01$. Reference values (RV) indicate clinical normative values used at Miura Medical Clinic.
